# Physical, Textural, Rheological, and Sensory Characteristics of Amaranth-Based Wheat Flour Bread

**DOI:** 10.1155/2020/8874872

**Published:** 2020-11-27

**Authors:** Sahreen Nasir, Farhana Mehraj Allai, Murtaza Gani, Shaiq Ganaie, Khalid Gul, Aabida Jabeen, Darakshan Majeed

**Affiliations:** ^1^Awantipora Department of Food Technology, Islamic University of Science & Technology, Pulwama, J&K, India; ^2^Department of Chemistry, KLDAV PG College Roorkee, Affiliated to HNB Garhwal University, Uttrakhand, India; ^3^Department of Food Science & Technology, SKUAST-K, Shalimar, J&K, Srinagar, India; ^4^Department of Food Science & Engineering, College of Agriculture and Biology, Shanghai Jiao Tong University, Shanghai, China

## Abstract

In this study, the chemical composition, colour analysis, and antioxidant properties of flour and bread were analysed. We also examined the rheological properties of dough and proximate, colour, textural, and organoleptic properties of amaranth wheat bread. Wheat flour was replaced by amaranth flour (AF) at 0-15% levels (100 : 0, 95 : 5, 90 : 10, and 85 : 15, respectively). AF supplementation increased the moisture (31.06 to 33.24%), ash (0.92 to 1.51%), protein (12.17 to 13.11%), fat (2.16 to 2.77%), and crude fibre content (1.11 to 1.72%) of the bread while the nitrogen-free extract and alkali water retention capacity decreased from 52.58 to 47.65% and 136.00 to 112.02%, respectively. The antioxidant activity evaluated by DPPH, FRAP, and total phenolic content was reduced with increased levels of AF. A significant impact on the physical properties like the weight of bread (increased from 474.00 to 489.30 g), height (went down from 80.00 to 74.33 cm), loaf volume (decreased from 1580.00 to 1518.30 cm^3^), and specific volume (reduced from 3.32 to 3.10 cm^3^ g^−1^) was observed with the replacement of wheat flour. Textural measurement depicted that hardness, chewiness, gumminess, springiness, and cohesiveness increased with the substitution of amaranth flour. Rheological parameters like complex viscosity, loss modulus, and storage modulus were also observed in all dough samples. Bread samples with 5%, 10%, and 15% of AF showed lower yellowness (*b*∗) and higher lightness (*L*∗) and redness (*a*∗) values for crust colour while lower *L*∗ and higher *a*∗ and *b*∗ values for crumb colour. The bread prepared by replacing 5% and 10% of AF is nutritionally as well as sensorially acceptable.

## 1. Introduction

Amaranth is a well-known pseudocereal because of its nutraceutical properties. Consumption of this grain helps in improving kidney complaints, constipation, and anaemia. Medicinally, its extracted water is used to treat pain in limbs, tumours, and wounds (Grubbens and Denton 1998). They also have anticancer and antioxidant properties [1]. Amaranth grain can be milled into flour by being popped, toasted, or extruded and therefore can be used as it is or consumed in other cereal products like crackers, cake, bread, pancakes, crepe, noodles, or any other bakery products [2]. Amaranth, as a key component of bakery items, enhances their antioxidant properties. They include several bioactive compounds, polyphenols, anthocyanins, tocopherols, and flavonoids [3]. The nutritional profile of this seed makes it appropriate to be blended with other cereal by-products to increase significantly the protein, dietary fibre, and mineral content [4–6]. Amaranth flour is gluten-free, so it can be blended with wheat flour to make composite flour-based products like bread to enhance the nutritional quality [3]. However, introduction of amaranth flour (AF) into wheat flour (WF) for bread making may lead to modifications of dough rheology and some changes in chemical, physical, and sensory properties [7].

Therefore, the aim of our study was to examine the antioxidant activity and chemical composition of flour, rheological properties of dough, and physical, textural, colour, and sensory properties of amaranth wheat bread.

## 2. Materials and Methods

### 2.1. Materials

Amaranth seeds were purchased from a local market in Jammu, India. The grains were cleaned manually to remove dust and other contaminants. Wheat flour, sugar, salt, vegetable oil, gluten, improver, and yeast were purchased from a local market in Srinagar, India. All the ingredients were stored separately in an airtight storage box at room temperature (25°C) for further analysis. All the chemicals used in this study were of Analytical Reagent (AR) grade.

### 2.2. Preparation of Amaranth Flour

Amaranth flour was prepared using a laboratory-grade grinder (M/S Philips, New Delhi, India). The seeds were ground to a fine powder and passed through a 177 *μ*m sieve to obtain flour of uniform size.

### 2.3. Antioxidant Activity of Flour

The total phenolic content of amaranth flour was evaluated using the modified method of Singleton & Rossi [8], DPPH by the method of Goupy et al. [9], and FRAP by Stratil et al. [10].

### 2.4. Proximate Composition of Flour and Bread

Standard methods of AOAC [11] were used to determine moisture (AOAC-993.26), ash (AOAC-923.03), fat (AOAC-2003-05), and protein (AOAC-960.52) content and nitrogen-free extract (NFE) of wheat flour (WF) and amaranth flour (AF). Alkaline water retention capacity (AWRC) of the samples was determined according to the procedure followed by [12].

### 2.5. Rheological Measurements

The rheological properties of the sample were measured by using a rheometer (Anton Paar, GmbH, Austria). After kneading, 7 g of the dough sample with 30 g of yeast added for formulation of bread was kept in-between the plates having parallel plate geometry with a diameter of 40 mm and a gap of 2 mm. The unwanted dough was removed gently, and after a relaxation period of 35 min, the measurements were taken. An oscillation test is the first step in which dough is not damaged when the frequency is increased from 0.1 to 10 Hz at a strain of 0.2%. The dough was further analysed for the creep recovery test for 280 s at a pressure of 110 Pa. Once pressure was removed, there was again relaxation time of 280 s. Nondestructive forces were used to measure different rheological behaviour. Measurements were taken thrice, and average measurements of all the samples did not exceed 5%; otherwise, all the measurements were repeated.

### 2.6. Product Development

#### 2.6.1. Preparation of Bread

Breads were prepared by mixing wheat flour and amaranth flour at three different levels (5, 10, and 15%) labelled as A5, A10, and A15 as presented in Table 1. A planetary mixer (Model SM-25, SINMAG, Japan) was used to mix all the ingredients together for 2 min at 214 rpm and fermented for 10 min. The contents were mixed to get an elastic and smooth dough. Thereafter, the dough was moulded and proofed for another 30 min followed by baking in an oven (deck oven, 601T, SINMAG, Japan) at 225°C for about 30 min. After baking, the bread loaves were kept at room temperature for cooling for 2 hrs and then sliced.

#### 2.6.2. Physical Evaluation of Bread

*(1) Height (mm)*. To determine the height (*H*), three slices of bread were taken from the middle of the loaf and placed edge to edge. The height of three slices of bread was measured by a scale in mm. The slices of bread were rotated at an angle of 90° for duplicate reading. This was repeated thrice, and the average height was taken in millimeters.

*(2) Loaf Volume (cm3) and Specific Volume Index (cm3/g)*. Loaf volume and specific volume of bread samples were measured by the rapeseed displacement method as described by See et al. *[*13*]* and Feili *et al. [*14*]*.

#### 2.6.3. Texture Analysis

The hardness, cohesiveness, springiness, chewiness, and gumminess of bread were measured using a texture analyser (TA.HDplus, Stable Micro Systems, Godalming, Surrey, UK) as per the standard method of AACC [15]. A sample of size 2.5 × 2.5 cm was taken from the middle of the bread and placed under the cylinder probe ((p/36 cylinder probe (36 mm)) to make a flat surface at all time. The compression test was done by using a load cell of 5 kg, 1.6 mm/s test speed, cycle number 2, compression distance 25%, and force 0.05 N.

#### 2.6.4. Colour Analysis

The samples used for the colour analysis of the bread crumb and crust (*L*∗, *a*∗, and *b*∗) were cut into cubes of 2 × 2 × 2 cm and placed in a digital colourimeter (Chroma Meter CR 300, Konica Minolta, Japan).

#### 2.6.5. Organoleptic Evaluation

Organoleptic evaluation of different bread samples was carried out as described by See et al. [13]. Breads were evaluated on the basis of colour (crust and crumb), texture (crust and crumb), aroma, taste, appearance, and overall acceptability on a 9-point hedonic scale, wherein 9 = like extremely and 1 = dislike extremely. Accordingly, all judges were trained and familiar with the quality parameters of bread.

### 2.7. Statistical Analysis

Data, with three replications (*n* = 3), were analysed using one-way analysis of variance. Duncan's multiple range test with significance defined at *p* ≤ 0.05 was used to compare the means. The statistical analysis was performed using SPSS (16.0, Chicago, IL).

## 3. Results and Discussion

### 3.1. Proximate Composition of Wheat and Amaranth Flour

The chemical compositions of wheat flour (WF), amaranth flour (AF), and their different mixtures are shown in Table 2. Amaranth flour was found to have high crude protein, crude fat, crude fibre, moisture, and ash content in comparison with wheat flour.

The protein content increased significantly with the increased levels of incorporation of amaranth flour due to higher protein content in amaranth flour as these are rich in some essential amino acids than whole wheat flour [16]. The fibre and ash content increased by increasing the whole amaranth flour as it contains bran which is a rich source of dietary fibres and minerals. However, during milling in refined wheat flour, bran/germ is removed, and thus, no fat content was found in refined wheat flour. Moreover, fat content increased with the increase in different substitution levels of whole amaranth flour [4, 17]. On the other hand, with the increase in the substitution level of amaranth flour, there was a significant decrease in NFE, AWRC, and moisture content as amaranth flour has less moisture content that justifies its suitability for long-term storage without deterioration [18].

### 3.2. Antioxidant Activity

The antioxidant activity of wheat flour (WF), amaranth flour (AF), and their different mixtures is shown in Table 3. The values of TPC decreased significantly (*p* ≤ 0.05) with the increase in the percentage level of amaranth flour. Similar decreases in DPPH and FRAP were also observed [19].

### 3.3. Colour Analysis

The colour measurements of the composite bread substituted with different levels of amaranth flour are depicted in Table 4. The results obtained for colour analysis of wheat flour, amaranth flour, and different substitution levels of amaranth flour showed that there were a decrease in lightness (*L*∗) value and an increase in redness and yellowness (*a*∗ and *b*∗) values with the increase in the percentage of amaranth flour. Similar observations were recorded by Sanz-Panella et al. [20].

### 3.4. Rheology of Dough

**Figure 1:**
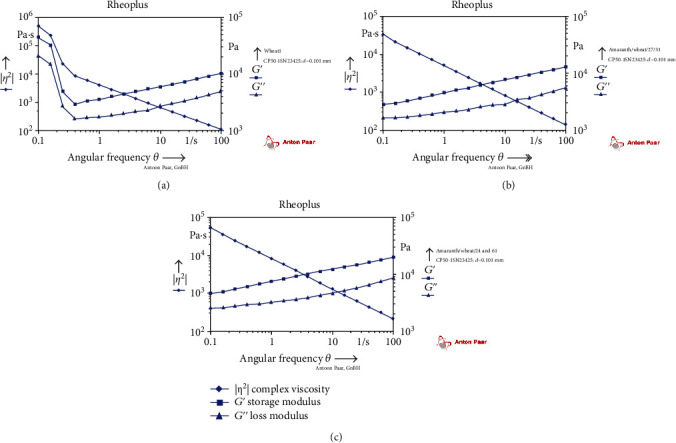
Plots showing variations in *G*′ and *G*^″^ as a function of angular frequency for a range of (a) 5% amaranth flour+95% wheat flour; (b) 10% amaranth flour+90% wheat flour; (c) 15% amaranth flour+85% wheat flour.

The rheological properties were measured in terms of the variation of storage modulus (*G*′), loss modulus (*G*^″^), and complex viscosity (*G*∗) and are shown in Table 5. The properties depend on the interaction between starch granules and their rigidity during the heating process. The results of the frequency sweep test were carried on dough samples, amaranth flour, and control. In all dough samples, the elastic modulus (*G*′) was higher than the viscous modulus (*G*^″^) as depicted in Figure 1. This might be due to the elastic-like behaviour of the dough. The complex modulus increased due to the interaction between the fibre structure and the wheat proteins. Similar results were found by Houben *et al. [*21*]*.

### 3.5. Proximate Composition of Bread

The proximate analysis of bread prepared with varying levels of wheat flour replaced with amaranth flour is presented in Table 6. Supplemented bread exhibited progressive increment in moisture content that suggests that amaranth starch granules have higher water absorption capacity than wheat starch flour [13]. Similar results were observed by Sanz-Panella et al. [20] and Ho et al. [22]. Higher ash content directly affected the bread quality as amaranth flour is high in mineral content. Table 6 shows that as the level of substitution increased, crude fibre and fat content increased significantly from 1.11% to 1.72% and 2.16% to 2.77%, respectively, due to the substitution of amaranth flour as it has higher lipids and dietary fibre content [5, 23]. The protein content of breads increased with the increase in the AF content as it is rich in essential amino acids and contains easily digestible proteins like albumins and globulins which considered amaranth flour highly nutritive [16].

Nitrogen-free extract (NFE) and alkali water retention capacity (AWRC) significantly decreased (*p* ≥ 0.05) from 52.58% to 47.65% and 136.00% to 112.02%, respectively. This decrease with the increase in the substitution level of amaranth flour could be attributed to the difference in quantitative distribution of protein fractions and physicochemical attributes of WF and AF [24, 25].

### 3.6. Physical Evaluation of Bread

The effect of amaranth flour on quality of bread by analysing various physical properties is shown in Table 7. Results depicted that with the increase in the level of AF (5-15%), the weight of composite bread increases from 474.00 g to 489.30 g, as AF has high water absorbing capacity and thus results in heavy dough due to low air entrapment [26]. The increasing percentage of AF in the bread samples significantly decreased the height of bread samples. The minimum height was observed in 15% amaranth flour when compared to the control. This decrease in bread height could be due to the low gluten content in the blends [27].

The volume of baked product and volume of bread loaf decreased significantly at higher concentration of amaranth flour, as the supplementation of wheat flour with nonglutinous flour reduces the gluten content and thus lowers the bread volume [28]. On the other hand, Sanz-Panella *et al.* [20] described this phenomenon as there is a high amount of water that results in gluten dilution, physical interactions, and chemical reactions among fibre components, and thus, during mixing, fermentation, and baking steps, the gluten matrix formation is affected.

### 3.7. Texture Profile Analysis

Texture parameters (hardness, cohesiveness, gumminess, chewiness, and springiness) are depicted in Table 8. Hardness is the foremost important parameter that shows the maximum force required to compress the composite bread and is usually showed by the first peak in the graph. The deviation in the force is due to the variation in the different substitution levels. The harder the bread, the higher the force required as the moisture level is low and there is the interaction between the gluten and fibrous material present in AF [29]. As there is an increase in the level of AF from 5 to 15%, the springiness of bread increased from 0.60 mm to 0.65 mm due to the presence of gelatinized starch and dough having gluten which produces more elastic dough and thus can form a continuous sponge structure of bread after baking [30]. Amaranth flour contains a higher amount of fat that entraps the air bubbles and thus causes porosity which increases the springiness in samples. The results are in accordance with Alvarez-Jubete et al. [19].

It is observed that by supplementation of AF in the bread, cohesiveness, gumminess, and chewiness increased due to the increase in hardness that results in crumbling of crumb and is usually associated with the loss of water which in turn is determined by the retrogradation properties of wheat flour [31].

### 3.8. Sensory Evaluation

**Figure 2:**
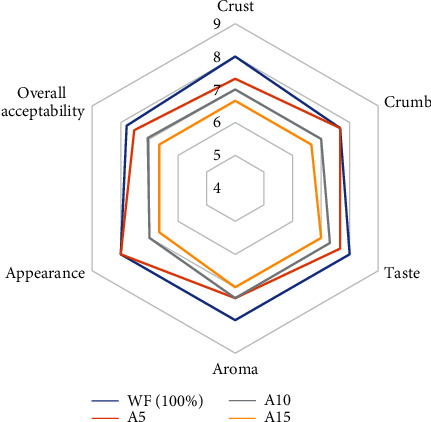
Sensory radar chart showing colour, texture, aroma, taste, and overall acceptability of bread substituted with various percentages of amaranth flour (AF). A5: 5% amaranth flour+95% wheat flour; A10: 10% amaranth flour+90% wheat flour; A15: 15% amaranth flour+85% wheat flour.

Sensory evaluation is an important parameter for analysing the quality of amaranth bread to meet the consumer requirements. The results are based on a 9-point hedonic scale to measure sensory characteristics of bread like colour, taste, texture, aroma, appearance, and overall acceptability of product [32]. Bread supplemented with amaranth flour showed significant (*p* ≤ 0.05) effect on all parameters and is shown in Table 9. The mean score for bread colour (crust and crumb) decreased significantly with the addition of amaranth flour. The highest mean score was noted for control (crust: 8.00 and crumb: 7.66) followed by 5%, 10%, and 15% substitution of amaranth flour as shown in Figure 2. This decrease in crust and crumb colour might be due to the similar colour of AF and whole wheat flour, and during baking, these reducing sugars caramelized to cause dark brown colour of bread samples [18]. The best texture for bread crust and crumb was observed in 10% amaranth flour. The effect could be due to the incorporation of AF that decreases the formation of the gluten network that failed to retain vapours produced, and therefore, the symmetry of bread decreased linearly with the increase in the concentration of amaranth. These results are in alignment with Emire and Arega [18].

The statistically significant difference was observed in aroma and taste. The mean score for aroma and taste decreases from 7.33 to 6.00 and 8.00 to 7.02, respectively, with the increase in addition of amaranth flour. This might be due to the nutty flavour of composite bread produced at a high temperature which could be objectionable in some bakery products. A similar observation was presented by Ayo [23]. The result analysed that overall palatability of supplemented bread was acceptable at 10% supplementation in terms of different physical attributes. It decreases linearly with the increase in concentration of amaranth flour and eventually resulting in the rejection of 15% amaranth-based bread [23].

#### 3.8.1. Colour Analysis

Table 9 shows the parameter *L*∗, *a*∗, and *b*∗ values for crust and crumb colour of bread. Amaranth flour causes significant changes in the crumb and crust colour of bread. In general, when AF concentration was raised, the tristimulus colour values (*L*∗, *a*∗, and *b*∗) in both crumb and crust were affected. Lightness and redness of bread crust increased by increasing the amaranth content in bread from 50.16 to 54.21 and 16.20 to 18.45, respectively, due to progressive loss of moisture content and oxidation of fats to produce hydrogen peroxide which oxidizes or bleaches pigment materials and results in increase in opacity ([20]), while yellowness for bread crust decreased as bran is present and amaranth flour has a darker colour than refined wheat flour. For the bread crumb, lightness decreased while redness and yellowness increased with the increased percentage of amaranth flour [32].

#### 3.8.2. Texture

The results pertaining to the mean score for crust and crumb texture are depicted in Table 10. The mean score for crust texture in all the treatments and control samples decreased significantly (*p* ≥ 0.05) from 8.00 to 6.66. The decrease in the texture of amaranth-based wheat flour bread is due to the incorporation of amaranth flour that decreases the formation of the gluten network that failed to retain vapours produced, and therefore, the symmetry of bread decreased linearly with the increase in concentration of amaranth. These results are in alignment with Emire and Arega [18].

#### 3.8.3. Taste and Aroma

Table 10 revealed that the taste score for amaranth-based bread ranges from 7.66 to 7.00. The decrease in the taste score during storage might be due to the staling of bread. These results are in-line with the findings of Emire and Arega [18].

The sensory score for aroma decreased significantly during storage. The aroma score of amaranth-based bread ranges from 8.00 to 7.00. The maximum mean value score for aroma was found in *T*_0_ (8.00) at 0 days of storage, and the minimum score of aroma was found in *T*_3_ (7.00) at the 6^th^ day of storage. This might be due to the nutty flavour of amaranth being pronounced with the increase in the concentration of amaranth flour. The results are in agreement with the findings of Ayo [23].

#### 3.8.4. Appearance and Overall Acceptability

The results pertaining to the score for appearance is depicted in Table 10. The overall acceptability of bread was found to decrease linearly with the increase in the concentration of amaranth and eventually resulting in the rejection of *T*_3_ [23].

## 4. Conclusion

Amaranth flour could be used as a replacement for wheat flour in the formulation of bread, enhancing the nutritional value and fibre content of the product. In this study, two different flours were substituted, viz., wheat flour and amaranth flour (up to 15%), to examine the changes in physicochemistry, total phenolic content, colour evaluation, physical parameters, texture profile analysis, and sensory attributes of wheat amaranth bread. Results demonstrated that the bread made with 10% amaranth flour had high levels of moisture content 32.60%, 12.96% protein,1.36% fibre, 2.61% fat, and 1.33% ash as compared to the control. Further, *L*∗ value increased while *a*∗ and *b*∗ values decreased in bread which is in the ratio of 90 : 10. Amaranth flour substitution resulted in the highest total phenolic content in the control (7.08) of all breads. All in all, using two different flours in the production of amaranth-based breads can lead to better products with higher nutrient content. However, a combination of flours could maintain the dough rheology. From the entire work carried out for the development of amaranth-based wheat flour bread, it can be concluded that the panellist preferred *T*_2_ (90 : 10) treatment in terms of organoleptic properties. Thus, the inclusion of amaranth flour in bread could be limited, not only for maintaining the product quality but also for preserving the benefits of nutritional ingredients

## Figures and Tables

**Table 1:** Formulation of wheat amaranth composite bread.

Ingredients	*T*_0_ (control)	*T*_1_ (A5)	*T*_2_ (A10)	*T*_3_ (A15)
Wheat flour (g)	2000	1900	1800	1700
Amaranth flour (g)	0	100	200	300
Yeast (g)	30	30	30	30
Sugar (g)	32	32	32	32
Salt (g)	20	20	20	20
Oil (ml)	200	200	200	200
Gluten (g)	20	20	20	20
Improver (g)	10	10	10	10
Water (ml)	1200	1200	1200	1200

*T*_0_ = 100% wheat flour (WF); *T*_1_ (A5) = 5%AF + 95%WF; *T*_2_ (A10) = 10%AF + 90%WF; *T*_3_ (A15) = 15%AF + 85%WF.

**Table 2:** Proximate composition of amaranth flour, refined wheat flour, and their different substitution.

Parameters	Wheat flour (100%)	Amaranth flour (100%)	A5+95% wheat flour (%)	A10+90% wheat flour (%)	A15+85% wheat flour (%)
Moisture (%)	12.36 ± 0.05^a^	7.94 ± 0.33^e^	11.71 ± 0.05^b^	11.21 ± 0.33^c^	10.44 ± 0.19^d^
Ash (%)	0.71 ± 0.02^e^	2.83 ± 0.03^a^	0.82 ± 0.05^d^	1.06 ± 0.19^c^	1.22 ± 0.03^b^
Protein (%)	12.04 ± 0.44^e^	13.85 ± 0.29^b^	12.37 ± 0.27^d^	12.76 ± 0.14^c^	13.10 ± 0.16^a^
Crude fibre (%)	0.75 ± 0.01^e^	4.60 ± 0.21^a^	0.92 ± 0.20^d^	1.02 ± 0.11^c^	1.17 ± 0.22^b^
Crude fat (%)	1.90 ± 0.43^e^	6.53 ± 0.33^a^	1.97 ± 0.05^d^	2.13 ± 0.19^c^	2.27 ± 0.19^b^
NFE (%)	72.24 ± 0.21^a^	64.25 ± 0.11^e^	74.39 ± 0.03^c^	73.63 ± 0.01^b^	73.30 ± 0.03^d^
AWRC (%)	122.00 ± 0.19^a^	110.00 ± 0.38^d^	118.00 ± 0.30^b^	111.00 ± 0.30^c^	103.00 ± 0.33^e^

Values are expressed as means of triplicate samples ± standard deviation (*n* = 3). Values with the same superscripts in a column are statistically similar while values with different superscripts are significantly different (*p* ≤ 0.05).

**Table 3:** Antioxidant activity of wheat flour (WF), amaranth flour (AF), and mixtures.

Parameters	Wheat flour (100%)	Amaranth flour (100%)	A5+95% refined wheat flour (%)	A10+90% refined wheat flour (%)	A15+85% refined wheat flour (%)
TPC (mg/g)	6.22 ± 0.05^a^	3.05 ± 0.33^c^	6.14 ± 0.05^a^	5.87 ± 0.33^b^	5.81 ± 0.19^b^
DPPH (*μ*mol Trolox/g)	3.80 ± 0.31^a^	3.18 ± 0.55^b^	3.08 ± 0.14^c^	2.87 ± 0.33^d^	2.83 ± 0.60^d^
FRAP (mg Trolox/g)	157.01 ± 0.58^a^	48.01 ± 0.85^e^	141.00 ± 0.65^b^	125.00 ± 0.33^c^	103.51 ± 0.94^d^

Values are expressed as means of triplicate samples ± standard deviation (*n* = 3). Values with the same superscripts in a column are statistically similar while values with different superscripts are significantly different (*p* ≤ 0.05).

**Table 4:** Colour analysis of wheat flour (WF), amaranth flour (AF), and mixtures.

Parameters	Wheat flour (100%)	Amaranth flour (100%)	A5+95% refined wheat flour (%)	A10+90% refined wheat flour (%)	A15+85% refined wheat flour (%)
*L*∗	79.91 ± 0.20^e^	91.63 ± 0.07^a^	87.26 ± 0.01^b^	84.41 ± 0.01^c^	81.92 ± 0.02^d^
*a*∗	2.76 ± 0.06^a^	0.41 ± 0.05^e^	0.57 ± 0.05^d^	0.73 ± 0.02^c^	0.82 ± 0.01^b^
*b*∗	16.55 ± 0.08^a^	9.41 ± 0.12^e^	9.84 ± 0.07^d^	10.04 ± 0.04^c^	10.45 ± 0.02^b^

Values are expressed as means of triplicate samples ± standard deviation (*n* = 3). Values with the same superscripts in a column are statistically similar while values with different superscripts are significantly different (*p* ≤ 0.05).

**Table 5:** Effect of amaranth wheat blended dough on rheological properties.

Treatments	Storage modulus (*G*′) (Pa)	Loss modulus (*G*^″^) (Pa)	Complex viscosity (*G*∗) (Pa s)
*T*_0_ (control)	9.07 × 10^3^	4.95 × 10^3^	9.38 × 10^2^
*T*_1_ (A5)	9.38 × 10^3^	5.60 × 10^3^	8.26 × 10^2^
*T*_2_ (A10)	9.30 × 10^3^	8.66 × 10^3^	9.15 × 10^2^
*T*_3_ (A15)	9.73 × 10^3^	7.97 × 10^3^	9.94 × 10^3^

*T*_0_ = 100% wheat flour (WF); *T*_1_ (A5) = 5%WF + 95%WF; *T*_2_ (A10) = 10%AF + 90%WF; *T*_3_ (A15) = 15%AF + 85%WF.

**Table 6:** Effect of substitution of amaranth flour on proximate composition of bread.

Parameters	Wheat flour (100%)	A5+95% wheat flour	A10+90% wheat flour	A15+85% wheat flour
Moisture (%)	31.06 ± 0.07^aD^	32.00 ± 0.03^aC^	32.60 ± 0.20^aB^	33.24 ± 0.13^aA^
Ash (%)	0.92 ± 0.61^dD^	1.14 ± 0.04^dC^	1.33 ± 0.05^dB^	1.51 ± 0.04^dA^
Protein (%)	12.17 ± 0.09^dD^	12.61 ± 0.05^dC^	12.96 ± 0.07^dB^	13.11 ± 0.03^dA^
Crude fibre (%)	1.11 ± 0.06^dD^	1.27 ± 0.06^dC^	1.36 ± 0.05^dB^	1.72 ± 0.05^dA^
Crude fat (%)	2.16 ± 0.01^dD^	2.32 ± 0.15^dC^	2.61 ± 0.36^dB^	2.77 ± 0.12^dA^
NFE (%)	52.58 ± 0.01^aA^	50.66 ± 0.02^aB^	49.41 ± 0.01^aC^	47.65 ± 0.02^aD^
AWRC (%)	136.00 ± 0.51^aA^	122.03 ± 0.16^aB^	116.01 ± 0.60^aC^	112.02 ± 0.51^aD^

Values are expressed as means of triplicate samples ± standard deviation (*n* = 3). Values with the same superscripts in a column are statistically similar while values with different superscripts are significantly different (*p* ≤ 0.05).

**Table 7:** Effect of substitution of amaranth flour on physical evaluation of bread.

Parameters	Wheat flour (100%)	A5+95% wheat flour	A10+90% wheat flour	A15+85% wheat flour
Weight (g)	474.00 ± 5.00^dD^	482.62 ± 1.15^cC^	488.00 ± 5.19^bB^	489.30 ± 3.05^aA^
Height (mm)	80.00 ± 0.01^aA^	76.00 ± 0.01^bB^	75.66 ± 1.15^cC^	74.33 ± 0.57^dD^
Loaf volume (cm^3^)	1580 ± 10.00^aA^	1551.6 ± 7.60^bB^	1543.3 ± 15.20^cC^	1518.3 ± 7.60^dD^
Specific volume (cm^3^/g)	3.32 ± 0.04^aA^	3.21 ± 0.01^bB^	3.19 ± 0.03^cC^	3.10 ± 0.04^dD^

Data represents the mean ± standard deviation (*n* = 3). Means followed by capital letter superscripts in a row differ significantly (*p* ≤ 0.05), and means followed by the same small letter superscripts in a column differ significantly (*p* ≤ 0.05).

**Table 8:** Effect of texture profile analysis (TPA) on hardness, cohesiveness, gumminess, chewiness, and springiness of bread substituted with amaranth flour during storage.

Parameters	Wheat flour (100%)	A5+95% wheat flour	A10+90% wheat flour	A15+85% wheat flour
Hardness (N)	2.10 ± 0.03^dB^	3.93 ± 0.04^cB^	4.22 ± 0.07^bB^	4.83 ± 0.06^aB^
Cohesiveness	0.80 ± 0.02^dD^	0.82 ± 0.02^cD^	0.84 ± 0.05^bD^	0.86 ± 0.03^aD^
Gumminess	1.10 ± 0.01^dC^	1.72 ± 0.03^cC^	2.05 ± 0.03^bC^	2.39 ± 0.02^aC^
Chewiness (kg/mm)	4.86 ± 0.04^dA^	7.05 ± 0.05^cA^	8.12 ± 0.02^bA^	9.36 ± 0.01^aA^
Springiness (mm)	0.60 ± 0.05^dD^	0.61 ± 0.01^cD^	0.62 ± 0.04^bD^	0.65 ± 0.07^aD^

Data represents the mean ± standard deviation (*n* = 3). Means followed by capital letter superscripts in a row differ significantly (*p* ≤ 0.05), and means followed by the same small letter superscripts in a column differ significantly (*p* ≤ 0.05).

**Table 9:** Effect of substitution of amaranth flour on colour of bread.

	Crust colour			Crumb colour		
	*L*∗	*a*∗	*b*∗	*L*∗	*a*∗	*b*∗
Wheat flour (100%)	50.16 ± 0.43^dD^	16.20 ± 0.32^aD^	34.21 ± 0.21^bA^	73.31 ± 0.20^dA^	1.56 ± 0.31^aD^	19.25 ± 0.05^aD^
A5+95% wheat flour	52.10 ± 0.20^dC^	17.47 ± 0.06^aC^	33.58 ± 0.09^bB^	69.65 ± 0.06^dB^	1.75 ± 0.08^aC^	19.82 ± 0.05^aC^
A10+90% wheat flour	54.83 ± 0.22^dB^	17.99 ± 0.08^aB^	33.08 ± 0.06^aC^	68.90 ± 0.54^dC^	1.88 ± 0.04^aB^	20.09 ± 0.07^aB^
A15+85% wheat flour	54.21 ± 0.31^dA^	18.45 ± 0.09^aA^	32.24 ± 0.07^aD^	67.43 ± 0.57^dD^	1.96 ± 0.03^aA^	21.78 ± 0.05^aA^

Data represents the mean ± standard deviation (*n* = 3). Means followed by capital letter superscripts in a row differ significantly (*p* ≤ 0.05), and means followed by the same small letter superscripts in a column differ significantly (*p* ≤ 0.05).

**Table 10:** Effect of substitution of amaranth flour on texture, taste, aroma, appearance, and overall acceptability of bread.

Parameter	*T*_0_ (control)	*T*_1_ (A5)	*T*_2_ (A10)	*T*_3_ (A15)
Crust	8	7.33	7	6.66
Crumb	7.66	7.66	7	6.66
Taste	8	7.66	7.31	7
Aroma	8	7.33	7.33	7
Appearance	8	8	7	6.66
Overall acceptability	7.8	7.53	7.06	6.66

*T*_0_ = 100% wheat flour (WF); *T*_1_ (A5) = 5%AF + 95%WF; *T*_2_ (A10) = 10%AF + 90%WF; *T*_3_ (A15) = 15%AF + 85%WF.

## Data Availability

Data are available as tables and figures.

## Additional Points

*The Manuscript Highlights*. (i) Baking properties of the pseudocereals as potential healthy and high-quality ingredients in breads were investigated. (ii) Experimental results showed better physical and textural properties and antioxidant activities of bread. (iii) Significant differences were obtained in the acceptability of the pseudocereal-containing gluten-free breads in comparison with the control. (iv) Developed breads with higher nutritional and fibre contents may serve as alternatives to normal breads.
